# Anaesthetic Management of Critical Tracheal Obstruction Secondary to Granulation Tissue in a Patient With an In Situ Tracheal Stent: A Case Report

**DOI:** 10.7759/cureus.86578

**Published:** 2025-06-23

**Authors:** Chooi Lin Koh, Sandy Lim, Andy Jian Kai Chua, Rayan Alsuwaigh, Chi Ho Chan

**Affiliations:** 1 Department of Anaesthesiology, Sengkang General Hospital, Singapore, SGP; 2 Department of Otorhinolaryngology, Sengkang General Hospital, Singapore, SGP; 3 Department of Respiratory and Critical Care Medicine, Changi General Hospital, Singapore, SGP; 4 Department of Anaesthesiology, Singapore General Hospital, Singapore, SGP

**Keywords:** acute respiratory failure (arf), airway stent, emergency airway management, tracheal obstruction, tracheal stenosis

## Abstract

Tracheal stenosis is a rare condition that poses significant challenges in airway management. We report a case of failed emergency awake fiberoptic intubation necessitating difficult front-of-neck access in a patient with respiratory failure, tracheal stenosis, and an in situ tracheal stent. The patient presented to the emergency department with acute respiratory distress and was urgently transferred to the operating theatre for emergent intubation. An awake intubation technique was attempted; however, the patient deteriorated during airway topicalization, necessitating rescue airway maneuvers. Bag-valve-mask ventilation proved difficult, and an attempt to insert a Portex™ size 6 endotracheal tube (Smiths Medical, Minneapolis, MN, USA) was unsuccessful due to resistance encountered after passing the vocal cords. An emergent tracheostomy was performed, but was complicated by the presence of the tracheal stent. Ultimately, a Shiley™ cuffless size 4 tube (Medtronic, Mansfield, MA, USA) was successfully inserted. Bronchoscopic assessment revealed granulation tissue both proximal and distal to the tracheal stent, with near-complete obstruction of the left main bronchus, accounting for the intubation resistance and ventilatory difficulties. The silicone tracheal stent remained in place with no signs of migration post-intubation. This case underscores the complexities of airway management in critical tracheal stenosis secondary to granulation tissue with an in situ tracheal stent.

## Introduction

Tracheal stenosis is a rare condition that presents significant challenges in airway management. The incidence of tracheal stenosis resulting from post-intubation injury is estimated to be approximately 1 in 200,000 individuals annually [1]. Tracheal stent placement is a technique utilized for the management of tracheal stenosis. However, tracheal stents can provoke local inflammation and growth of granulation tissue [2], which has been reported in between 11.5% and 74% of patients [2-5]. The presence of an in situ tracheal stent and the formation of granulation tissues may pose significant challenges to airway management. In the context of secondary infection or mechanical irritation, granulation tissue formation can cause progressive or sudden central airway obstruction (CAO) and may remain clinically silent until critically severe.

We present a case of failed emergency awake fiberoptic intubation requiring difficult front-of-neck access in a patient with respiratory failure, tracheal stenosis, and an in situ tracheal stent. This report highlights the challenges in airway management of critical airway stenosis secondary to granulation tissue with tracheal stenting in situ. Management includes the restoration of the airway and targeted treatment of granulation tissue. This article adheres to the CARE (CAse REport) guidelines and institutional ethical guidelines for the publication of case reports. The patient provided written consent for publication.

## Case presentation

A 71-year-old female with an in situ tracheal stent for tracheal stenosis presented to the emergency department with acute breathlessness. Her medical history included Guillain-Barré syndrome diagnosed at the age of 19, which required prolonged intubation and was complicated by tracheomalacia. The severity of the tracheomalacia necessitated the insertion of a long-term tracheostomy tube for over 47 years. Over time, she developed tracheal stenosis and required serial dilation and the insertion of a silicone tracheal stent via rigid bronchoscopy approximately five years prior to the current presentation, followed by decannulation of the tracheostomy tube a year after. Her condition was stable leading up to the current presentation.

**Figure 1:**
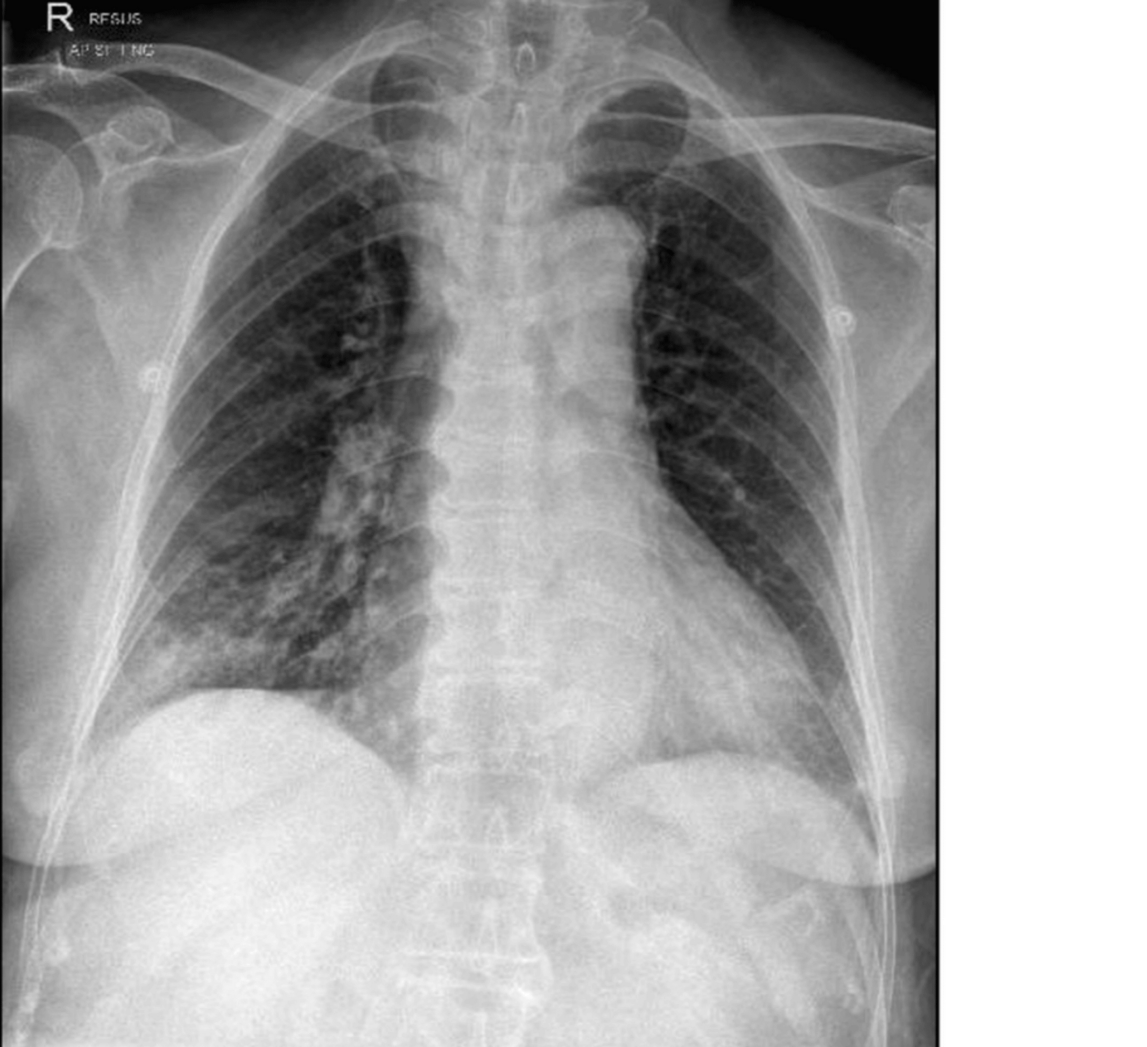
Chest X-ray image on presentation demonstrating patchy airspace opacification projected over the right lower zone. The tracheal stenosis was not well appreciated.

The patient was admitted to the emergency department with a four-day history of progressively worsening dyspnea and fever. Upon initial assessment, she was found to be in significant respiratory distress, with an oxygen saturation of 71% on room air. She exhibited stridor and a productive cough yielding copious amounts of sputum. She was administered oxygen via face mask at 8 L/min, resulting in an improvement in SpO_2_ to 98%. Arterial blood gas analysis indicated type 2 respiratory failure, with a partial pressure of carbon dioxide of 54.8 mmHg and a partial pressure of oxygen of 96 mmHg while on 8 L/min of oxygen via face mask. Although her chest X-ray revealed a right lower lobe consolidation consistent with community-acquired pneumonia, there was a high suspicion of CAO, given her presentation with stridor, a history of tracheal stenosis with a tracheal stent in situ, and type 2 respiratory failure, as shown in Figure 1. She was evaluated by the otolaryngology (ENT) team, and a bedside endoscopic examination was performed, revealing no obstructing lesions from the oral cavity to the vocal cords. Her previous tracheostomy site had narrowed down to a pinpoint hole, which appeared to be contiguous with her trachea, as there was a tiny amount of air flow with respiration, but this was inadequate for respiratory needs. At this point, it was clear that the patient required ventilatory support, but routine orotracheal intubation would potentially be difficult due to a suspected obstruction below the level of the larynx. After discussion with the intensive care team, the decision was made to transfer her to the operating room for an awake fiberoptic bronchoscopic assessment of the trachea and endotracheal intubation.

In the operating room, an Ambu® aScope™ 4 Broncho Slim 3.8/1.2 bronchoscope (Ambu A/S, Ballerup, Denmark) was prepared for fibreoptic bronchoscopy assessment of the trachea and intubation. In anticipation of a difficult airway and narrowed tracheal diameter, a McGrath™ MAC video laryngoscope (Medtronic, Heerlen, Netherlands), endotracheal tubes (ETTs) of various sizes, including microlaryngeal tubes, and laryngeal mask airways (LMAs) were prepared. An ENT surgeon was present in the operating room, scrubbed and ready in case a surgical airway was required. Airway topicalization was performed using a 2% lidocaine mouth gargle and 10% xylocaine spray to the mouth and posterior pharynx. A transtracheal injection of 0.5 mL of 2% lidocaine was administered via the previously created semi-closed tracheostomy. During the topicalization of the vocal cords with 2% lidocaine using the spray-as-you-go technique, the patient exhibited drowsiness and significant desaturation, subsequently becoming unresponsive within seconds. The awake intubation procedure was abandoned. An attempt at bag-valve-mask ventilation was unsuccessful. Rapid sequence induction was initiated with propofol 60 mg and rocuronium 30 mg. Additional small boluses of propofol were administered as needed to achieve adequate ventilation, with a total propofol dose of 110 mg given. Endotracheal intubation was performed using a Portex™ size 6 ETT (Smiths Medical, Minneapolis, MN, USA), which was successfully inserted between the vocal cords on the first attempt but could not be further advanced to the correct position due to significant resistance in the trachea. The patient was able to be ventilated with an improvement in SpO_2_ to 100% (up from the lowest levels of 30%), but this was achieved only with elevated airway pressures (35-40 cm H_2_O). General anaesthesia was maintained with sevoflurane at a minimum alveolar concentration of 0.7 once adequate ventilation was established. The patient remained hemodynamically stable throughout the procedure, with no requirement for inotropic or vasopressor support.

**Figure 2:**
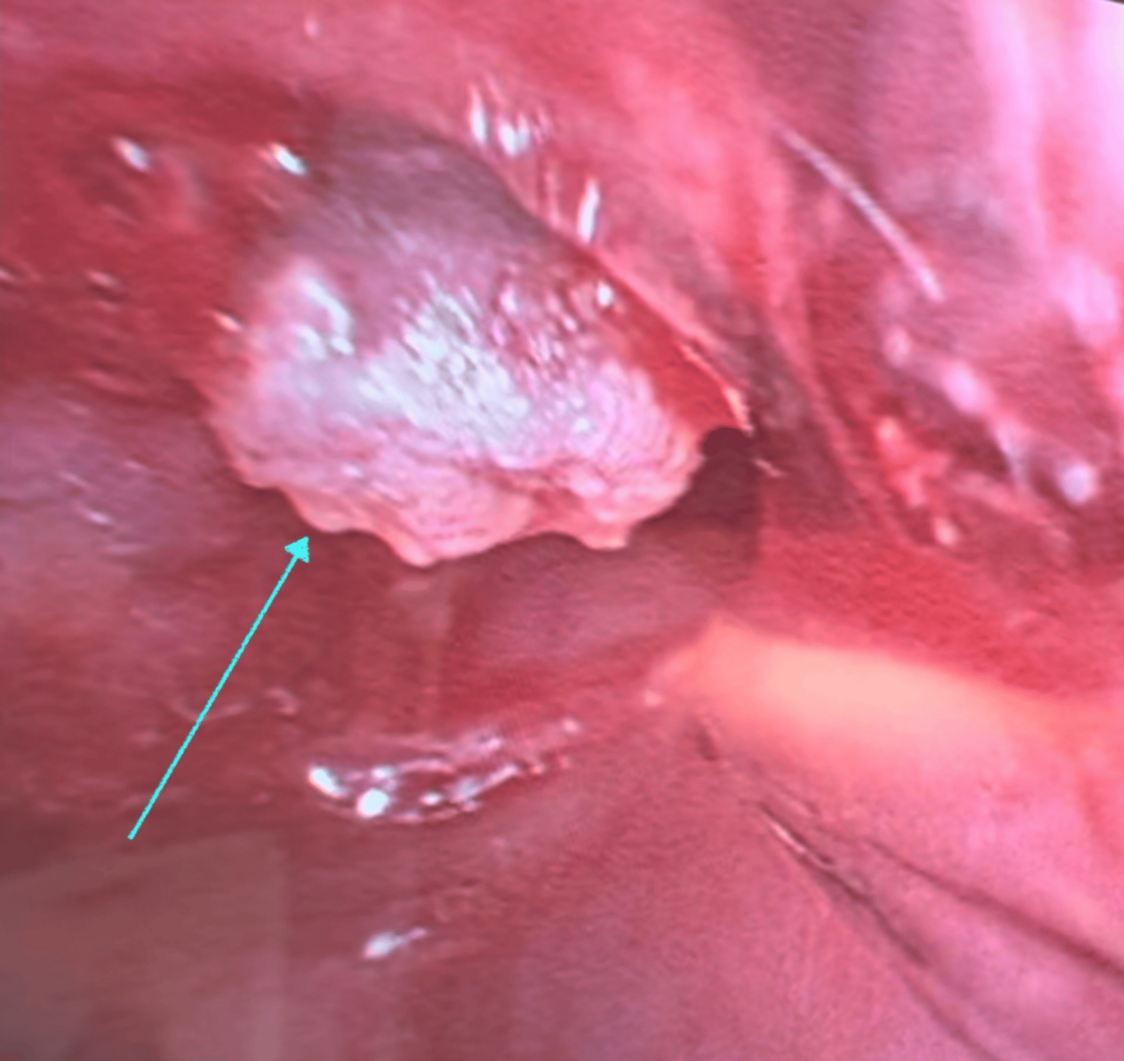
Fibreoptic bronchoscopy view through the endotracheal tube showing granulation tissue proximal to the tracheal stent, indicated by the cyan arrow.

**Figure 3:**
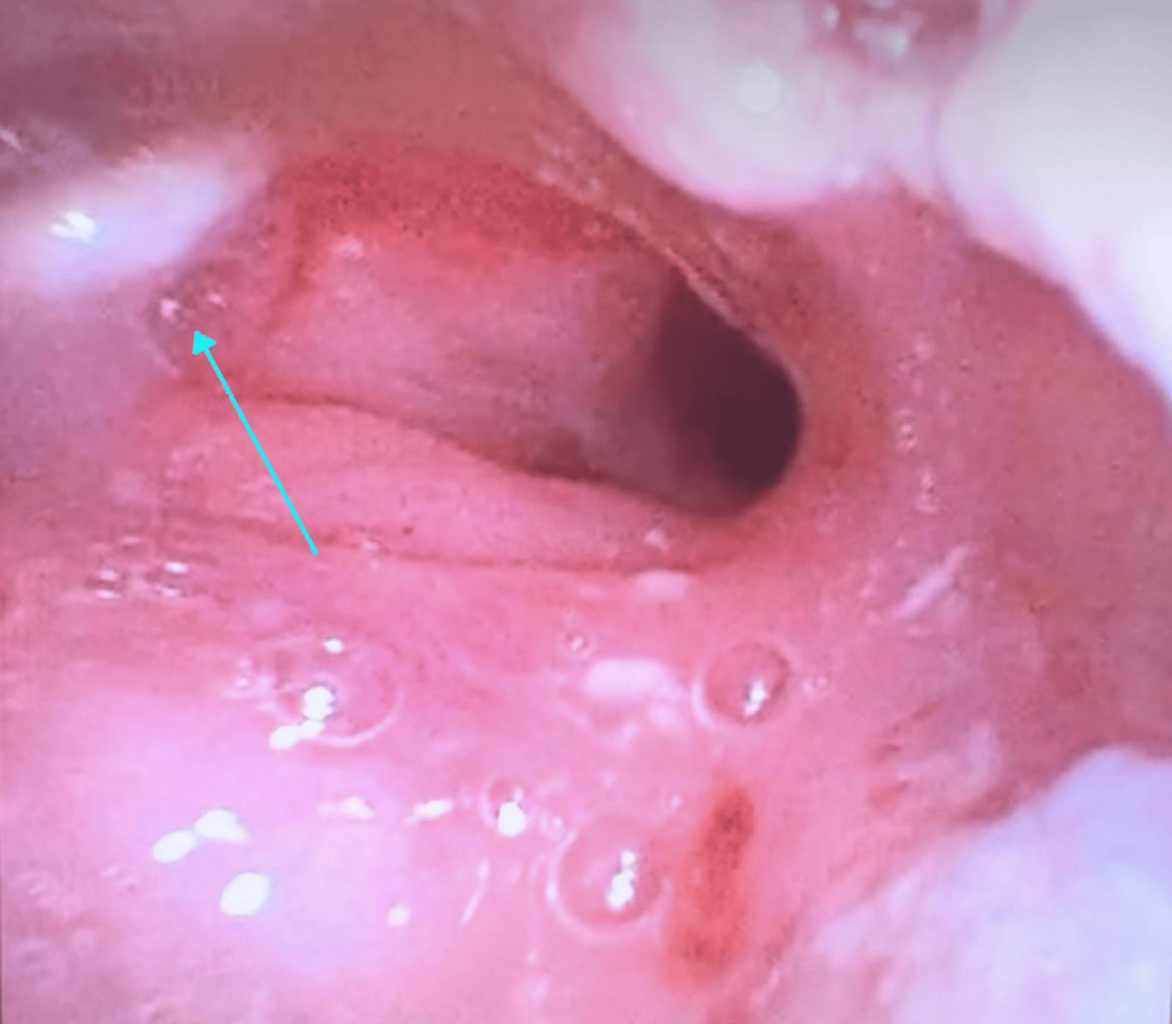
Fibreoptic bronchoscopy view through the tracheostomy tube revealing granulation tissue distal to tracheal stent, causing near-complete obstruction of the left main bronchus with a slit-like orifice that remained intermittently patent, indicated by the cyan arrow.

An attempt was made to pass a flexible endoscope through the ETT, but no additional meaningful assessment could be made of the distal airway as the view was severely obscured by secretions that could not be suctioned out without causing rapid desaturation. Given that the airway remained tenuous with a sub-optimally positioned ETT and persistently elevated airway pressures, the decision was made to proceed with an urgent tracheostomy. The creation of the tracheostomy proved challenging. A wide incision was made through the ex-stoma site, including the pin-hole defect. There was heavy scar tissue encountered during the subcutaneous and deep soft tissue dissection. As the tracheal incision was made, a large amount of firm granulation tissue was encountered, preventing the insertion of a standard size 7 tracheostomy tube. Ultimately, a Shiley™ size 4 cuffless tracheostomy tube (Medtronic, Mansfield, MA, USA) was inserted successfully, resulting in significant improvement in ventilation and a reduction in peak airway pressures. After securing the tracheostomy tube, the airway was re-assessed using the fiberoptic bronchoscope. Bronchoscopy through the ETT revealed extensive granulation tissue at its distal tip, just proximal to the tracheal stent, likely contributing to the resistance encountered during its advancement and preventing smooth insertion of the ETT, as shown in Figure 2. Additionally, bronchoscopy via the tracheostomy tube showed granulation tissue obstructing the distal trachea and extending into the left main bronchus (Figure 3 and Video 1), with a slit-like orifice that remained intermittently patent. Despite this, equal air entry was heard bilaterally on auscultation, suggesting partial preservation of airflow to the left lung. The ETT was removed, and the patient was transferred to the intensive care unit (ICU) for further management.

**Video 1:** Fibreoptic bronchoscopy view through the tracheostomy tube revealing granulation tissue distal to tracheal stent, causing near-complete obstruction of the left main bronchus.

**Figure 4:**
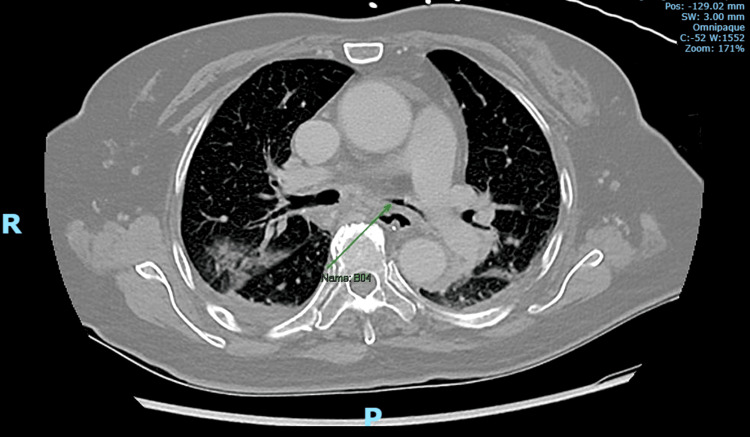
Computed tomography image showing soft tissue thickening around the trachea and bilateral main bronchi, with narrowing of the bronchi lumen, more prominent on the left (labelled B04).

In the ICU, empiric antibiotic therapy was continued for the treatment of community-acquired pneumonia, with a good clinical response. Computed tomography (CT) of the thorax demonstrated consolidation in the right lower lobe with cystic bronchiectasis, and a smaller focus of consolidation with surrounding ground-glass changes in the right upper lobe. In addition, soft tissue thickening around the trachea and bilateral main bronchi with associated luminal narrowing was observed, as shown in Figure 4. The patient’s condition improved, and she was transferred to the high dependency unit after four days and subsequently to the general ward on the eighth postoperative day.

**Figure 5:**
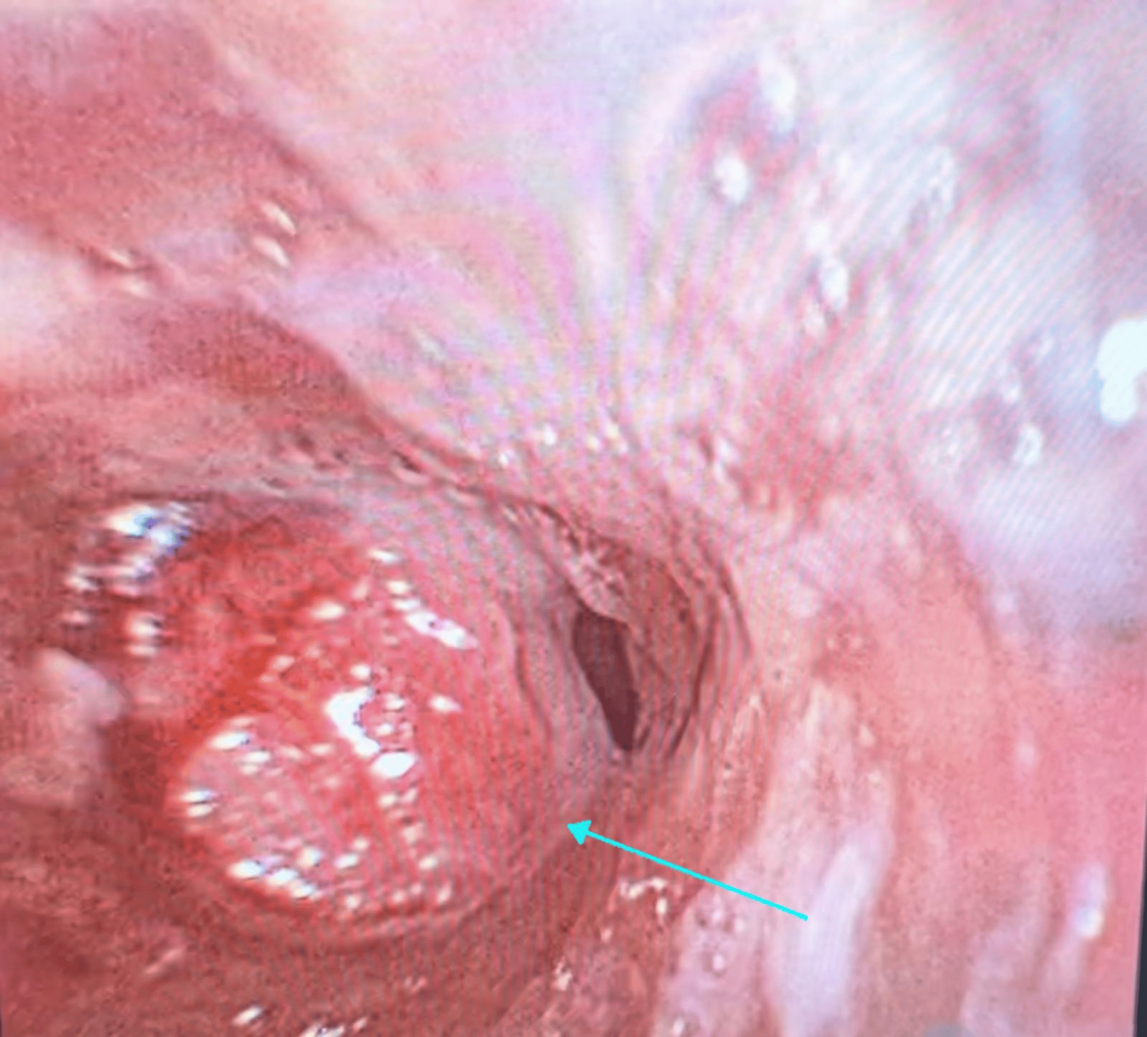
Fibreoptic bronchoscopy view through the tracheostomy tube revealing granulation tissue distal to tracheal stent, causing complete left main bronchus obstruction and partial right main bronchus obstruction, indicated by the cyan arrow.

However, on postoperative day 11, she developed acute respiratory distress due to partial dislodgement of the tracheostomy tube. She was unable to tolerate reinsertion of the tracheostomy tube at the bedside and was therefore transferred to the operating room for tracheostomy tube reinsertion under general anaesthesia. Tracheostomy tube reinsertion in the operating room was uneventful; however, following the procedure, air entry into the left lung was noted to be limited, accompanied by intermittent episodes of desaturation. Effective ventilation of the left lung was only achievable intermittently. An intraoperative flexible bronchoscopy performed by the respiratory team revealed approximately 90% obstruction of the tracheal lumen by granulation tissue at the distal end of the tracheal stent, with only a small crescent-shaped gap noted posteriorly, as shown in Figure 5 and Video 2. The left bronchus could not be assessed due to the obstruction, but the right lung appeared unremarkable. Eventually, ventilation of the left lung was restored when the tracheostomy tube was rotated slightly clockwise, suggesting a dynamic extrinsic compression component or pliable granulation tissue that responded to minimal positional shifts.

**Video 2:** Fibreoptic bronchoscopy view through the tracheostomy tube revealing granulation tissue distal to tracheal stent, causing complete left main bronchus obstruction and partial right main bronchus obstruction.

Given the nearly complete obstruction of the central airway and the risk of catastrophic airway collapse or bleeding during intervention, the patient was transferred to a tertiary referral centre following stabilization, where venovenous extracorporeal membrane oxygenation (ECMO) was instituted preoperatively to facilitate safe bronchoscopy and therapeutic debulking. Subsequently, cryo-debulking of granulomatous tissue was performed via rigid bronchoscopy, with hemostasis achieved using adrenaline, ice-cold saline, argon plasma coagulation, and laser therapy. This was followed by balloon bronchoplasty of the left and right main bronchi. ECMO support was successfully weaned off the day after the procedure. The patient had an uneventful recovery and was discharged three days after.

## Discussion

CAO is a potentially life-threatening emergency that increases airway resistance, leading to hypoventilation and respiratory failure. CAO is a recognized complication of airway stents, with reported issues including stent migration, tumor growth, infection, mucous plugging [4-6], and granulation tissue formation, particularly at the proximal and distal ends of the stent [7]. Initially, granulation tissues are soft and vascular, which may cause bleeding and airway obstruction [8]. As the granulation tissue matures, it becomes more fibrous and is covered by epithelium, resulting in tracheal narrowing [8]. Neo-epithelialization and granulation tissue formation after self-expandable metallic airway stent insertion can occur as early as three to six weeks [9], with a reported incidence of about 11.5%, of which 7.7% had obstructive granulomas [5]. This may be more common with the use of silicone stents and Montgomery T-tubes, with reported cases of up to 50% and 74%, respectively [2-4]. Lower respiratory tract infections in patients with tracheal stents also promote the formation of granulation tissue and decrease survival [3]. However, only 3-12% of patients develop clinically significant stenosis requiring intervention [10]. Granulation tissue can cause airway obstruction, obstruction of tracheostomy tube fenestrations, and difficulty in reinserting the tracheostomy tube in the event of accidental decannulation [8]. If granulation tissue causes CAO, emergent rigid bronchoscopy, balloon, ablation, and/or excision of granuloma tissue may be warranted to relieve the airway obstruction [11]. Other options may include stenting or surgical repair and anastomosis [11].

Airway management in patients with tracheal stents can be challenging. Depending on the underlying indication for tracheal stent insertion, some patients with tracheal malignancy may be exposed to chemotherapy and radiation therapy, complicating laryngoscopy [12]. The presence of a tracheal stent suggests narrowing of the airway, which can prevent the insertion of an adequately sized ETT. Additionally, granulation tissue growth at the proximal end of the stent can interfere with the advancement of the ETT, as in this case. Stent migration, influenced by risk factors such as stent size, length, location, type, design, and duration since placement, may also be precipitated by airway manipulation, ETT insertion, aggressive suctioning, elevated positive-pressure ventilation, and forceful coughing during emergence, resulting in catastrophic consequences [12,13]. Front-of-neck assess, the last-resort airway technique for unanticipated difficult airways, can be technically challenging when a tracheal stent is in situ. Moreover, surgical dissection can lead to stent damage, compression, or misplacement [14]. Therefore, it is of utmost importance to thoroughly assess patients with tracheal stents prior to airway manipulation; however, this can prove to be challenging in the emergency setting.

Diagnosing tracheal stenosis can be challenging. Tracheal stenosis may remain asymptomatic until the lumen diameter has been reduced by 50-75% [8]. Patients with significant stenosis may initially present with coughing, followed by dyspnea on exertion and difficulty in clearing secretions when the airway lumen diameter is less than 10 mm, and stridor with dyspnea at rest when the airway diameter is less than 5 mm [8]. An anteroposterior plain chest X-ray film may show irregularity in the trachea suggestive of tracheal stenosis [10], but this abnormality may not always be seen. Therefore, a high index of suspicion is imperative when attending to patients with a history of prolonged intubation, tracheostomy, or the presence of an airway stent. Further radiological evaluation of the airway for tracheal stenosis may include posteroanterior and lateral chest radiography, fluoroscopy, and CT [15]. However, silicone stents, depending on their composition, may be radiolucent on standard imaging, thereby limiting radiological assessment. Nonetheless, multi-detector CT is able to accurately detect 97% of complications identified by bronchoscopy, even in patients with silicone stents [16]. Magnetic resonance imaging (MRI) is well suited for evaluating soft tissue structures such as granulation tissue; however, its image quality and diagnostic utility may be limited by artifacts arising from tracheal stents [17]. Clinical stents are manufactured from various materials, each with distinct MRI safety profiles. Nickel-titanium alloy stents typically do not undergo displacement and exhibit minimal heating and artifact production [17]. Stainless steel stents, though depending on the grade, are generally stable during MRI but may produce artifacts that compromise image clarity [17]. Silicone stents, although non-metallic and considered MRI-safe, can also degrade image quality by introducing artifacts that obscure adjacent anatomical structures, particularly in small-caliber airway imaging [18]. Ultimately, bronchoscopy is the gold standard for visualization of the tracheal airway and identification of any granulation tissue in patients with a tracheal stent. Given the insidious progression of granulation tissue and its potential for critical obstruction, routine bronchoscopic surveillance is essential in stented patients, especially after airway infections, prolonged secretions, or any new respiratory symptoms. Ultrasound assessment may also be useful for the localization of the tracheal stent and assessment of the tracheal stenosis [19].

When preparing for elective procedures, a thorough patient assessment would be useful for planning airway management. This includes taking a comprehensive history of the reason for stent insertion and the type of stent used, as well as evaluating the airway for stent position and patency, the presence of granulomas, and the presence of trachea-esophageal fistula [20]. Measurement of the narrowest tracheal diameter may aid in selecting an appropriately sized fibreoptic bronchoscope to avoid a "cork-in-bottle" scenario. An ENT surgeon should be present in the operating room and prepared for the need for rescue airway maneuvers prior to intubation. The use of high-flow nasal oxygenation should be considered for pre-oxygenation and apneic oxygenation to prolong safe apnea time, especially in patients with anticipated difficult intubations and bag-valve-mask ventilation.

Regarding airway technique, a supraglottic airway device would be the safest option to avoid tracheal intubation and should be used if it is feasible with the planned procedure and is not otherwise contraindicated [14]. If tracheal intubation is required, the fibreoptic technique may be the safest approach and should be performed if possible to ensure correct positioning of the ETT within or just above the stent and no stent migration occurs during the intubation process [14]. However, during emergency intubation, an awake fibreoptic intubation technique may not always be possible. If tracheal intubation was performed without fiberoptic bronchoscopy guidance, it is recommended to assess the integrity of the tracheal stent and the positioning of the ETT after tracheal intubation [14].

High-frequency jet ventilation may also be useful to provide oxygenation and gas exchange without the need for stent manipulation. This is particularly relevant in the context of therapeutic bronchoscopic procedures. In the airway management of therapeutic bronchoscopy for CAO, jet ventilation appears to have a comparable, if not lower, complication rate than spontaneous or volume-cycled ventilation [21]. Jet ventilation catheters may be positioned either proximal or distal to the stenosis. Distal placement is associated with lower distal tracheal pressures, more consistent oxygen delivery due to minimal entrainment, and more effective carbon dioxide clearance, and may therefore be preferred when it does not interfere with the planned procedure [22]. In addition, there is a theoretical concern that jet ventilation delivered proximal to a tracheal stent may induce stent dislodgement, although this is rare and has not been reported in the literature. However, caution is warranted in cases of severe CAO, as impaired exhalation, particularly when the airway diameter is less than twice that of the jet catheter, may result in elevated distal airway pressures and increase the risk of pneumothorax [22].

In the event of unanticipated difficult intubation or failed fibreoptic bronchoscopy-guided intubation, the existing difficult airway guidelines and recommendations do not include the management of patients with an in situ tracheal stent [23,24]. Nonetheless, the primary goal remains to establish alveolar oxygen delivery, and techniques including laryngoscopy, supraglottic airway devices, and bag-valve mask ventilation should still be attempted based on the actual clinical scenario. However, it is important to note that while front-of-neck access for a cannot-intubate-cannot-oxygenate scenario is feasible, it can be technically challenging, and the surgeon should be alerted to the presence of a stent early. Rigid bronchoscopy-guided percutaneous tracheostomy insertion has been described and can be considered [25,26]. ECMO should be considered early if available, especially in patients with severe airway stenosis [20].

In our case, ECMO was not available at our institution. Although a jet ventilator was available, it had not been pre-prepared and was therefore not feasible for use in the management of the acute deterioration. When the patient deteriorated during awake intubation, our plan B was to attempt endotracheal intubation, as ICU admission was anticipated for pneumonia management, and an LMA was suboptimal for prolonged ICU care. In hindsight, however, using an LMA as a bridging plan B could have been appropriate, allowing for low-skill fibreoptic intubation or tracheostomy creation once oxygenation and ventilation were secured. Nevertheless, we proceeded with endotracheal intubation, and we were able to achieve reasonably effective ventilation through the ETT. Tracheostomy was created thereafter.

## Conclusions

In conclusion, emergency airway procedures in patients with an in situ tracheal stent can be challenging. Thorough patient assessment and close communication with the surgical team are crucial to ensure a favourable outcome. Contingency plans should be in place for unanticipated difficult airways, with consideration of ECMO in high-risk patients. This case also highlights the absence of formal airway algorithms tailored to patients with in situ tracheal stents, underscoring the need for institutional protocols and multidisciplinary training for such high-risk scenarios.
